# Enhancing Polymer Sustainability: Eco-Conscious Strategies

**DOI:** 10.3390/polym16131769

**Published:** 2024-06-22

**Authors:** Aparna Beena Unni, Tomy Muringayil Joseph

**Affiliations:** 1 Faculty of Science and Technology, University of Silesia, 75 Pulku Piechoty 1a, 41-500 Chorzow, Poland; 2 Department of Polymer Technology, Faculty of Chemistry, Gdańsk University of Technology, G. Narutowicza, 80-233 Gdańsk, Poland; tomymuringayiljoseph@gmail.com

**Keywords:** polymers, biodegradable, sustainability, eco-friendly, recycling, circular economy

## Abstract

Polymer sustainability is a pressing concern in today’s world driven by the increasing demand for environmentally friendly materials. This review paper provides a comprehensive overview of eco-friendly approaches towards enhancing the sustainability of polymers. It synthesized recent research and developments in various areas such as green polymer synthesis methods, biodegradable polymers, recycling technologies, and emerging sustainable alternatives. The environmental impact of traditional polymer production processes and the importance of adopting greener alternatives were critically examined. The review delved into the advancements in polymer recycling technologies like mechanical, chemical, and biological processes aimed at minimizing plastic waste and promoting a circular economy. The innovative approaches such as upcycling, hybrid methods etc., which offer promising solutions for addressing plastic pollution and achieving long-term sustainability goals were also analyzed. Finally, the paper discussed the challenges and future prospects of eco-friendly approaches for polymer sustainability, emphasizing the need for researchers and concerted efforts from scientists across industries and academia to drive meaningful change towards a more sustainable future.

## 1. Introduction

**Figure 1:**
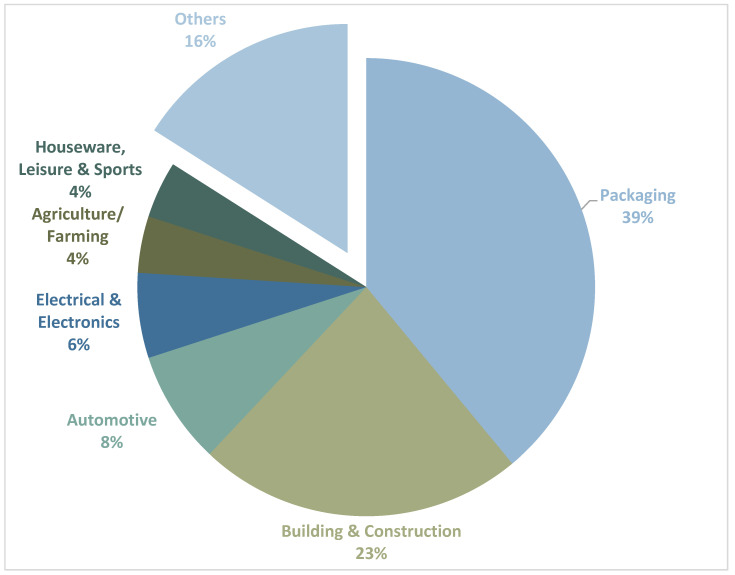
Pie chart showing the percentage use of plastics in different sectors of the European Union. Data derived from references [1,3,4].

Plastics have become an inevitable component of the present world due to their wide range of applications. Every year, over 300 million tons of plastics are manufactured globally for the consumer world [1,2]. The largest sector using plastics is packaging, which accounts for approximately 40% of total plastic consumption globally due to it being lightweight, durable, and an effective method of preservation for food as well as for the creation of goods made from plastics [1,3,4]. The second reported user is the construction sector, where the plastics are employed in applications such as pipes, insulation, flooring, and windows etc., due to their energy efficiency and low maintenance needs [5]. The automotive industry uses plastics for components like dashboards, bumpers, and fuel tanks, benefiting from their lightweight nature to improve fuel efficiency and reduce emissions [6]. Consumer goods, including household items, furniture, toys, and electronics, heavily rely on plastics for their cost efficiency and design flexibility [7]. In the medical field, plastics are crucial for manufacturing medical devices, packaging, disposable syringes, and prosthetics, due to their sterility and versatility [8,9] Besides, the electronics industry utilizes plastics for casings, circuit boards, and insulating components, leveraging their durability and electrical insulation properties [6,10]. Lastly, in agriculture, plastics enhance productivity through products like greenhouse films, irrigation systems, and mulching films [11,12,13]. Therefore, the usage of plastic is almost inevitable in the current scenario. To have a better picture, Figure 1 summarizes the percentage use of plastics in different sectors of the European Union.

Having discussed the importance of plastic usage, it is even more important to discuss the other side of the story, which are the downsides of plastic usage. Approximately 90.6% of the above-mentioned plastics are fossil fuel-based [4]. The remaining forms such as mechanically recycled plastics (postconsumer) constitute 8.9%; chemically recycled plastics (postconsumer) are less than 0.5%; and the rest such as bio-based plastics (including bio-attributed) are less than 0.1% [4].

Environmental pollution is the main issue created by fossil fuel-based plastics. It massively contributes to the waste which mostly ends up in landfills or natural environments like oceans or other water bodies persisting for hundreds of years and causing severe harm to marine life and ecosystems [14,15]. Its production process is highly energy-intensive, relying heavily on fossil fuels which leads to substantial greenhouse gas emissions, thereby exacerbating global warming and climate change [16,17]. This reliance also depletes nonrenewable resources such as petroleum and natural gas raising sustainability concerns and contributing to geopolitical tensions [6]. Another aspect is that the chemicals involved in the production, use, and disposal of fossil-based plastics pose serious human health risks, including endocrine disruption, reproductive harm, and cancer, affecting both the general population and workers in the industry [18]. The recycling of fossil-based plastics is inefficient and costly due to contamination and the mixing of different plastic types, resulting in only a small fraction being recycled while the majority is either landfilled or incinerated which further contributes to the environmental pollution [1].

The present generation is becoming well aware and vigilant of the environmental concerns and sustainability imperatives. As polymeric materials play a pivotal role in every aspect of modern life right from packaging and construction to electronics and transportation, the environmental impact of these materials has garnered heightened scrutiny [19,20,21,22]. The presence of plastics in our ecosystems, coupled with their persistence in the environment and contribution to global pollution, underscores the urgent need for innovative and eco-conscious strategies to mitigate their adverse effect [23,24,25]. 

**Figure 2:**
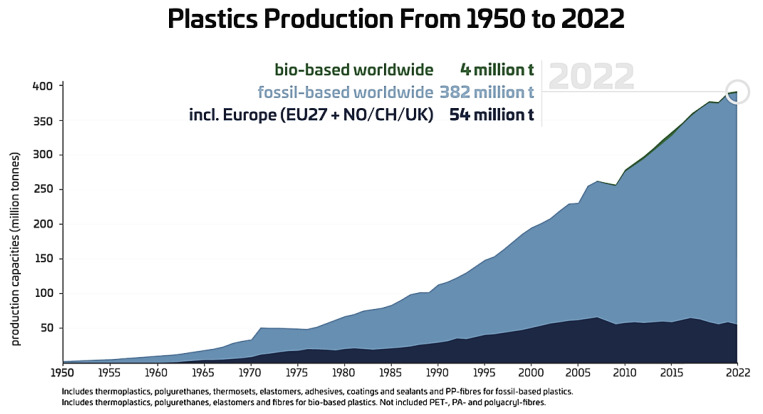
Plastics production from 1950 to 2022. (Source: https://renewable-carbon.eu/graphics, accessed on 1 June 2024) [26].

Figure 2 shows the growth in plastic production from the year 1950 to 2022. From the figure, one can observe a tremendous increase in the plastic with advancing time.

It is also notable that bio-based plastic production also advances with time. Hence, enhancing the polymer sustainability has emerged as a paramount objective [27,28]. 

Considering the practical implementations of sustainability, understanding the nuances between different types of polymers and the established standards that verify their environmental claims is crucial. The growing emphasis on sustainability has led to an increase in products and materials claiming to be “green” or environmentally friendly. However, confusion often arises regarding the authenticity of these claims due to varying standards and criteria used to evaluate sustainability [29]. One major area of confusion lies in the definitions and terminology, such as the difference between bio-based and biodegradable polymers. Bio-based polymers are derived from renewable resources, while biodegradable polymers are designed to break down in the environment [30]. Not all bio-based polymers are biodegradable and not all biodegradable polymers being bio-based can mislead the consumers [31]. Another issue is incomplete lifecycle assessments (LCAs). LCAs are essential for evaluating the environmental impact of polymers throughout their lifecycle, but inconsistent or partial assessments can result in misleading sustainability claims [32]. To address this confusion, established standards can provide guidelines for verifying green claims. ISO standards such as ISO 14040/14044 offer comprehensive guidelines for conducting LCAs, ensuring a thorough evaluation of environmental impacts at all stages of a product’s lifecycle [33]. ASTM standards like ASTM D6400 specify requirements for labelling plastics designed for composting, which helps to authenticate compostability claims of biodegradable polymers [34].

European standards like EN 13432 also play a crucial role by setting requirements for packaging recoverable through composting and biodegradation, ensuring that materials break down in composting conditions [35]. The USDA BioPreferred Program certifies products with significant bio-based content, making it easier for consumers to identify products made from renewable resources [36]. Besides, the Global Recycled Standard (GRS) certifies products containing recycled content, ensuring traceability and transparency in the supply chain to validate recycled content claims and reduce environmental impact [37].

In response to the plastic crisis, there has been a growing interest in developing sustainable alternatives and innovative approaches to polymer production, use, and disposal. On this aspect, this article explored the diverse array of eco-conscious strategies available for enhancing polymer sustainability, encompassing a spectrum of approaches ranging from material innovations and recycling technologies to circular economy principles and regulatory interventions. By providing a comprehensive overview of these strategies, we aimed to throw light on the complexities of polymer sustainability and elucidate the opportunities and challenges inherent in the pursuit of a more sustainable future with polymers.

The key central factors for polymer sustainability are biodegradable alternatives, bio-based polymers, and advanced recycling technologies, each offering unique pathways to reducing the environmental impact of polymer materials. Biodegradable polymers hold promise for degrading naturally in the environment, alleviating the burden of plastic waste accumulation and pollution. They possess specific chemical and bonding properties that facilitate their breakdown in the environment through natural processes such as enzymatic action, hydrolysis, and photodegradation etc. [38]. The factors contributing to their biodegradability include the presence of easily hydrolyzable bonds such as esters, amides, and glycosidic linkages. These polymers often contain hydrophilic groups that absorb water which in turn enhance the hydrolytic degradation and in general, they possess lower crystallinity which makes them more accessible to water and enzymes [39]. Bio-based polymers, derived from renewable resources such as plant biomass offer a sustainable alternative to fossil fuel-derived plastics, thereby reducing carbon emissions and mitigating resource depletion. After addressing bio-based polymers, the advancements in recycling technologies, including mechanical recycling, chemical recycling, and upcycling present opportunities to close the loop on polymer waste; transforming discarded materials into valuable resources etc., will be discussed. 

## 2. Biodegradable Polymers

As discussed earlier, the traditional polymer production processes based on petrochemical feedstocks have significant and multifaceted environmental impacts. The extraction and processing of these fossil fuels release substantial amounts of greenhouse gases, contributing significantly to climate change [40,41]. The energy-intensive nature of polymer production exacerbates carbon emissions. For example, a ton of polyethylene can emit between 1.8 to 3.2 tons of CO_2_ equivalent [1]. The manufacturing process also generates numerous hazardous byproducts and waste materials that can pollute air, water, and soil, leading to long-term environmental hazards [6]. Apart from this, the traditional polymers have extremely long degradation times leading to persistent pollution issues. Microplastics formed from the breakdown of larger plastic debris can infiltrate ecosystems and food chains, causing harm to wildlife and potentially posing risks to human health [42]. The pervasive nature of plastic pollution, coupled with the environmental costs of its production, underscores the urgent need for more sustainable practices in the polymer industry. 

**Figure 3:**
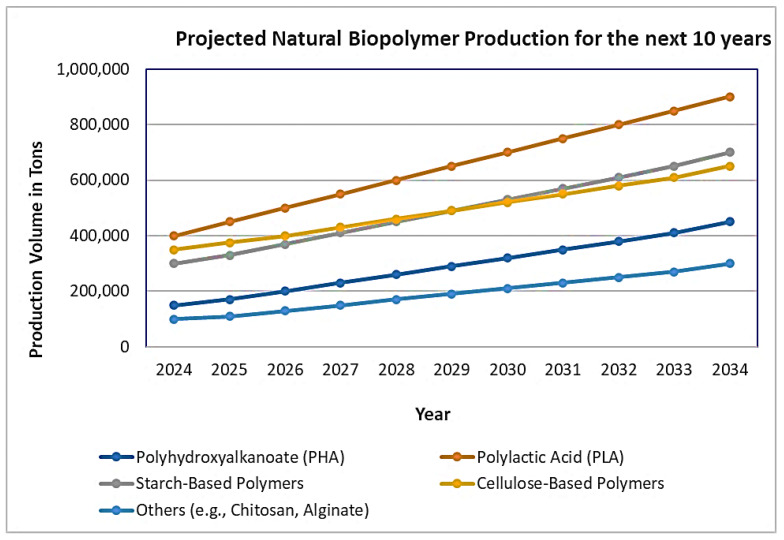
Projected annual production volumes of various natural biopolymers from 2024 to 2034 (in tons). Data derived from references [43,44].

Biodegradable polymers have gained significant attention due to their potential to reduce environmental pollution caused by traditional petroleum-based plastics. These polymers can be broken down by microorganisms into water, carbon dioxide (or methane), and biomass under natural conditions. There is now a significant increase in the production and usage of these polymers. A considerable increase in the implementation and shift to the usage of these products is expected in the future as well. Figure 3 shows the prediction of annual production volumes of various natural biopolymers from 2024 to 2034. The current market study forecasts annual growth of 17% for bio-based polymers between 2023 and 2028 [26].

The first biodegradable polymer, polyhydroxybutyrate (PHB), was developed by Maurice Lemoigne in 1926 [45]. He isolated PHB from the bacterium Bacillus megaterium and his pioneering work laid the foundation for the development and study of biodegradable polymers. Building on Lemoigne’s initial discovery, research into polyhydroxyalkanoates (PHAs) later gained momentum. These biopolymers, produced via bacterial fermentation of sugars and lipids, were recognized for their potential as sustainable alternatives to petrochemical-derived plastics [46].

**Figure 4:**
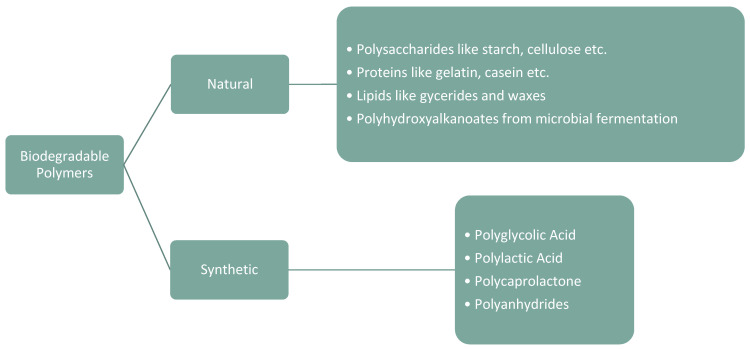
Biodegradable polymer major classifications.

Biodegradable polymers can be classified into three main categories based on their origin and production methods (Figure 4).

### 2.1. Natural Biodegradable Polymers 

Natural biodegradable polymers exhibit a wide range of properties like biocompatibility and biodegradability. Cellulose, chitosan, starch, and proteins such as collagen are among the most commonly studied natural polymers. These polymers possess inherent mechanical strength, flexibility, and thermal stability, making them suitable for diverse applications. 

### 2.2. Various Synthesis Methods

There are various methods employed for the synthesis of natural biodegradable polymers, including chemical modification, enzymatic reactions, and microbial fermentation etc. 

#### 2.2.1. Chemical Modification 

**Figure 5:**
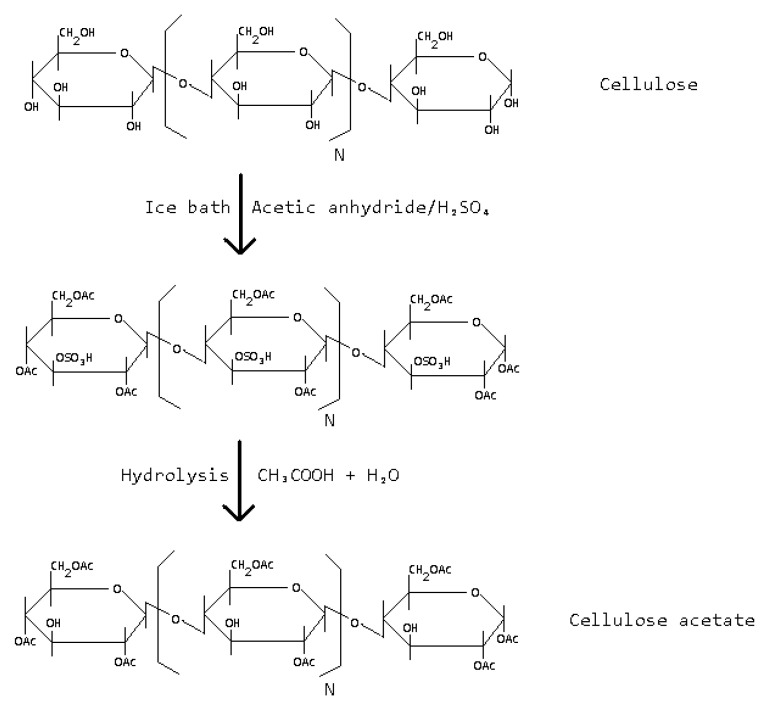
Cellulose acetate preparation [59].

Chemical modification involves altering the chemical structure of natural polymers through covalent bonding with functional groups or additives [47,48]. This method enhances the polymer’s properties such as mechanical strength, thermal stability, and solubility, expanding its potential applications. The most common modification strategies include esterification [49,50], etherification [51,52], acylation [53,54], grafting [55,56], and cross-linking [57,58]. For example, cellulose can be chemically modified to produce cellulose acetate, which exhibits improved mechanical properties and processability compared to native cellulose. Figure 5 shows the mechanism of the cellulose acetate preparation via chemical modification. 

#### 2.2.2. Enzymatic Reactions 

Enzymatic reactions offer precise control over polymer structure and functionality leading to controlled modifications without harsh reaction conditions or toxic byproducts. The enzymatic reactions typically occur under mild reaction conditions like ambient temperature and atmospheric pressure. This minimizes the energy consumption and environmental impact compared to conventional chemical methods. Enzymes that function under physiological pH and temperature ranges reduce the need for harsh solvents or reagents that can generate hazardous byproducts and waste streams.

#### 2.2.3. Microbial Fermentation Processes 

**Figure 6:**
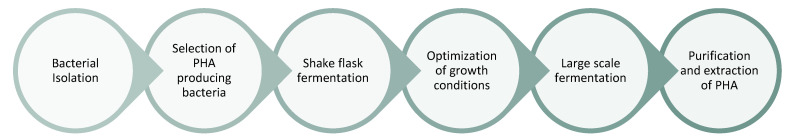
Schematic representation of PHA production steps.

Microbial fermentation processes utilize microorganisms to produce natural polymers with high purity and yield, contributing to sustainable manufacturing practices [60,61]. Microorganisms such as bacteria, fungi, and yeast are engineered to synthesize polymers using renewable carbon sources. Polyhydroxyalkanoates (PHAs) for example, are biodegradable polyesters produced via the microbial fermentation of renewable substrates like sugars or lipids. The process steps involved in PHA production are given in Figure 6. The process offers high yield and purity of polymers with tailored properties contributing to sustainable manufacturing practices.

Therefore, the natural biodegradable polymers offer environmental, economic, and societal benefits. It can be used as agricultural waste and byproducts, generating revenue for farmers and creating new markets. These polymers are safer and reduce exposure to harmful chemicals. With increasing consumer demand for eco-friendly products and stringent regulations, they align with global sustainability goals and promote a circular economy.

## 3. Synthetic Biodegradable Polymers

Synthetic biodegradable polymers are designed to degrade under environmental conditions, primarily via microbial activity, thus reducing their environmental footprint. Some of the key synthetic biodegradable polymers widely used are polyesters like:

*Polylactic Acid (PLA):* PLA is synthesized from renewable resources like corn starch and sugarcane through fermentation followed by polymerization [62,63]. It is known for its high mechanical strength and transparency, making it suitable for packaging, disposable tableware, and biomedical implants. However, its relatively low thermal stability and brittleness limit some applications [64].

*Polycaprolactone (PCL):* PCL is a semi-crystalline polymer produced via ring-opening polymerization of ε-caprolactone [65]. It is characterized by its low melting point, excellent biodegradability and compatibility with various biomedical applications, such as drug delivery systems and tissue engineering scaffolds [66].

*Polyhydroxyalkanoates (PHAs):* PHAs are microbial polyesters synthesized via bacterial fermentation of sugars and lipids [67]. They are fully biodegradable and can be tailored to exhibit a range of mechanical properties from rigid plastics to elastomers depending on their monomer composition. PHAs find applications in packaging, agricultural films, and medical devices [68].

*Nylon 4,6*: Polyamides like Nylon 4,6, where this aliphatic polyamide is known for its high melting point (~265 °C, 225 °C), strength, and rigidity [69]. It biodegrades more readily than traditional nylons, making it suitable for specialized applications where biodegradability is required [70]. There are also superior materials like polyetheretherketone (PEEK) having high-temperature stability and biocompatibility that can withstand continuous temperatures up to 250 °C and have a melting point of around 343 °C [71]. 

*Poly(sebacic acid)*: Yet another type is poly(sebacic acid) which are polyanhydrides synthesized via polycondensation of diacids and exhibit surface erosion during degradation, providing controlled release properties [72]. These polymers are primarily used in biomedical applications such as drug delivery systems. 

*Biodegradable polyurethanes*: There are also biodegradable polyurethanes that are made from bio-based monomers like polyols and diisocyanates [73]. The biodegradable polyurethanes combine flexibility, toughness, and biodegradability. They are used in medical devices, tissue engineering, and controlled-release applications [74]. 

*Polyvinyl alcohol*: Synthetic biodegradable polymers include water-soluble polymers like polyvinyl alcohol, PVA. It is a synthetic polymer that dissolves in water and degrades into nontoxic products. They are synthesized through the polymerization of vinyl acetate monomer followed by the hydrolysis (saponification) process resulting in the formation of polyvinyl acetate (PVA) [75]. It is used in applications requiring water solubility and biodegradability, such as laundry pods, packaging films, and medical applications.

### 3.1. Synthesis Methods

#### 3.1.1. Ring-Opening Polymerization (ROP) [76,77]

This method involves the polymerization of cyclic monomers like lactides and caprolactones, yielding polymers with controlled molecular weights and architectures. Catalysts such as tin(II) octanoate are often used to initiate the polymerization process.

#### 3.1.2. Polycondensation [78,79]

Polycondensation involves the step-growth polymerization of diacids and diols or diamines, producing polyesters, polyamides, and polyanhydrides. This method is commonly used for the synthesis of high molecular weight polymers with desired thermal and mechanical properties.

#### 3.1.3. Bacterial Fermentation [80,81]

This process utilizes genetically engineered bacteria to produce PHAs from renewable substrates. The fermentation conditions are optimized to maximize yield and tailor the polymer properties by varying the monomer composition.

#### 3.1.4. Reactive Extrusion [82,83]

Reactive extrusion integrates polymerization and processing in a single step, allowing for the continuous production of polymers. This method is particularly useful for producing blends and composites with enhanced properties. 

Both natural and synthetic biodegradable polymers play essential roles in addressing environmental concerns associated with plastic waste. Natural polymers offer the advantages of biocompatibility and renewable sourcing but face challenges in consistency and property customization. Synthetic polymers, on the other hand, provide versatility and precise control over material properties, albeit with potentially higher production costs and environmental considerations. The choice between these types of polymers depends on the specific application requirements, economic factors, and environmental impact considerations. Table 1 summarizes and compares the main points of biodegradable polymers.

## 4. Green Polymer Synthesis Methods

The environmental movements in 1970s raised awareness about pollution and resource depletion, prompting initial interest in sustainable practices including proposed development in polymer chemistry [84]. One of the major milestones in the practical implementations of environmentally friendly processes was the development of green chemistry principles by Paul Anastas and John Warner in the late 1980s [85]. They laid the groundwork for the systematic approach to reducing the environmental impact of chemical processes including polymer synthesis. A comprehensive framework was provided for designing environmentally friendly chemicals and processes for polymers. Yet another landmark in green polymer synthesis was the development of PLA as a biodegradable polymer. Although PLA was discovered earlier, its commercial production and refinement into a viable green polymer began in the late 1980s and 1990s by companies like NatureWorks [86]. They played a crucial role in developing processes to produce PLA from renewable resources, focusing on reducing environmental impact. Also, during this period, significant advancements with the introduction of supercritical CO_2_ polymerization were initiated. The usage of supercritical CO_2_ as a solvent could reduce the reliance on the then-used harmful organic solvents [87]. Concurrently, the development of solvent-free polymerization and the increased use of renewable resources such as natural polymers and bio-based monomers gained traction. The late 1990s and early 2000s witnessed the emergence of enzyme-catalyzed polymerization, offering mild and highly specific reaction conditions that minimized side reactions and energy consumption [88,89]. Photopolymerization techniques also advanced along with utilizing light to initiate polymerization without the need for solvents thereby reducing the emission of volatile organic compounds [90,91]. These pioneering efforts laid the groundwork for a diverse array of green polymer synthesis methods. It is continuously evolving and is driven by the ongoing research and technological innovations aimed at enhancing sustainability and reducing the environmental footprint of polymer production.

Some of the green polymer synthesis methods include: 

*Solvent-Free Polymerization techniques:* Solvent-free polymerization eliminates the use of volatile organic compounds which are commonly used as solvents in traditional polymer synthesis processes. Besides, it lowers production costs by eliminating the need for solvent procurement, handling, and disposal. The absence of solvents also means that the final polymer product is free of solvent residues, potentially enhancing its purity and performance. However, this method poses significant challenges in thermal control, as managing the heat generated during polymerization can be difficult especially for temperature-sensitive monomers or polymers. Likewise, the high viscosity of the reaction mixture can impede mass and heat transfer, affecting the polymerization rate and molecular weight distribution [87]. Techniques such as melt polymerization and solid-state polymerization fall under this category [92,93,94,95].

*Renewable Resources:* Using renewable resources for polymer synthesis involves materials derived from biomass, such as natural polymers (e.g., starch, cellulose, chitosan) and bio-based monomers (e.g., lactic acid, isosorbide). This approach offers substantial sustainability benefits by reducing reliance on finite fossil fuels and lowering the carbon footprint of polymer production [96]. Bio-based polymers are often biodegradable, addressing the growing concern over plastic waste. Their biocompatibility makes them suitable for applications in the medical and pharmaceutical fields. However, the inconsistent supply and quality of biomass can pose significant challenges to production stability. Besides, the modification of natural polymers to achieve desired properties can require complex and costly processing steps, which may hinder their widespread adoption [97].

*Enzyme-Catalyzed Polymerization:* Enzyme-catalyzed polymerization uses enzymes such as lipases, peroxidases, and laccases to catalyze polymer formation under mild conditions. This method is advantageous due to its environmental compatibility and the mild reaction conditions it requires, typically occurring at ambient temperatures and pressures, thereby reducing energy consumption [89]. Enzymes offer high selectivity, minimizing side reactions and resulting in polymers with well-defined structures. The challenge is in maintaining enzyme activity and stability during large-scale production. Besides, the high cost of enzyme production and purification can limit the economic viability of this method for large-scale applications [98].

*Photopolymerization:* Photopolymerization involves the use of light, usually ultraviolet (UV), to initiate polymerization. This method can be performed in solution or as a solvent-free process. Photo-initiators absorb light and generate reactive species that propagate polymerization [99,100]. One of the main advantages of photopolymerization is the precise spatial and temporal control it offers to the polymerization process. This control allows for patterning, making it particularly useful in applications such as 3D printing and microfabrication. Besides, photopolymerization is energy efficient due to its ambient temperature processing and significantly reduces VOC emissions when conducted as a solvent-free process. However, the penetration depth of UV light is limited, which can be problematic for thick or highly pigmented systems [101].

*Atom Transfer Radical Polymerization (ATRP):* The sustainability of some conventional methods is also reported to have considerable improvements. For example, Atom Transfer Radical Polymerization (ATRP) is a prominent controlled radical polymerization technique renowned for its precision in controlling polymer molecular weight and architecture. It involves the reversible activation and deactivation of a growing polymer chain through a redox process, which is typically mediated by a transition metal catalyst and a suitable ligand [102]. The sustainability of ATRP has been significantly enhanced through various green chemistry adaptations. Traditional ATRP processes often rely on organic solvents and higher levels of metal catalysts, which pose environmental and health risks. However, advancements such as water-based ATRP and the use of renewable solvents have mitigated these concerns. Water-based ATRP employs water as the solvent, which is not only environmentally benign but also cost-effective and safe [103]. Reducing the amount of metal catalyst through techniques like activators regenerated via electron transfer (ARGET) and initiators for continuous activator regeneration (ICAR) further aligns ATRP with green chemistry principles by minimizing the environmental footprint and toxicity of the process [104,105]. These innovations in ATRP technology not only enhance its environmental profile but also expand its utility in producing high-performance polymers from sustainable and renewable resources, contributing to the advancement of green polymer synthesis. 

*Ring-Opening Polymerization (ROP)*: ROP is a versatile polymerization technique particularly suited for the synthesis of biodegradable and bio-based polymers, such as polylactide (PLA) and polycaprolactone (PCL) [63,106,107]. Sustainable methods of ROP focus on using renewable monomers, green solvents, and environmentally friendly catalysts. Bio-based monomers like lactide, glycolide, and caprolactone are derived from renewable resources such as corn, sugarcane, and other biomass. These monomers undergo ROP to form polymers that are biodegradable to reduce long-term environmental impact. Green solvents, including supercritical CO_2_, ionic liquids etc., are increasingly used in ROP to replace traditional (often toxic) organic solvents. The development of metal-free organo-catalysts and enzymatic catalysts for ROP has significantly reduced the reliance on heavy metal catalysts which can be toxic and pose disposal challenges. These advancements not only improve the environmental footprint of ROP but also expand its applications in biomedical fields, where high purity and biocompatibility are essential.

*Click chemistry:* Sustainable methods in Click chemistry have emerged as powerful tools for green polymer synthesis due to their high efficiency, selectivity, and compatibility with various reaction conditions. Click reactions, particularly copper-catalyzed azide-alkyne cycloaddition (CuAAC) [108,109] and strain-promoted azide-alkyne cycloaddition (SPAAC), enable the rapid and reliable coupling of functional groups to form covalent bonds under mild conditions [110]. Click chemistry minimizes the need for harsh reagents and solvents, reducing waste generation and environmental impact. Efforts have been made to address these sustainability issues by developing greener variants of CuAAC, such as copper-free click reactions or the use of water as a solvent, which reduce the environmental impact of the reaction [111]. Furthermore, click reactions typically proceed in high yields, resulting in minimal byproducts and maximizing the utilization of starting materials. Green click chemistry approaches involve the use of bio-derived or renewable substrates and the development of catalysts that are nontoxic and easily recyclable [112,113]. These sustainable practices make click chemistry an attractive method for the synthesis of green polymers with tailored properties for applications in drug delivery, tissue engineering, and sustainable materials etc.

Methods like supercritical CO_2_ polymerization using supercritical CO_2_ as a green solvent [114] and microwave-assisted polymerization methods like solvent-free microwave polymerization, aqueous microwave polymerization [115,116], ionic liquid-assisted polymerization [117,118], and mechanochemical polymerization [119,120] etc., are also found to be effective considering the improvement of sustainability. The usage of green/biodegradable catalysts and initiators [121,122] and renewable initiators [123] etc., could also improve the sustainability. 

In conclusion, the green polymer synthesis methods represent a significant advancement in the field of polymer chemistry, aligning industrial practices with the principles of sustainability and environmental stewardship. Eliminating harmful solvents, utilizing renewable resources, and employing environmentally benign catalysts can significantly reduce the ecological footprint of polymer production. It could enhance the efficiency, specificity, and environmental compatibility of polymer synthesis. As research and innovation continue to drive the development of these green methods, they hold the promise of producing high-performance, sustainable polymers for a wide range of applications. The green technologies are not only crucial for mitigating environmental impact, but also for fostering a more sustainable future in polymer manufacturing.

## 5. Sustainable Polymer Recycling Technologies

**Figure 7:**
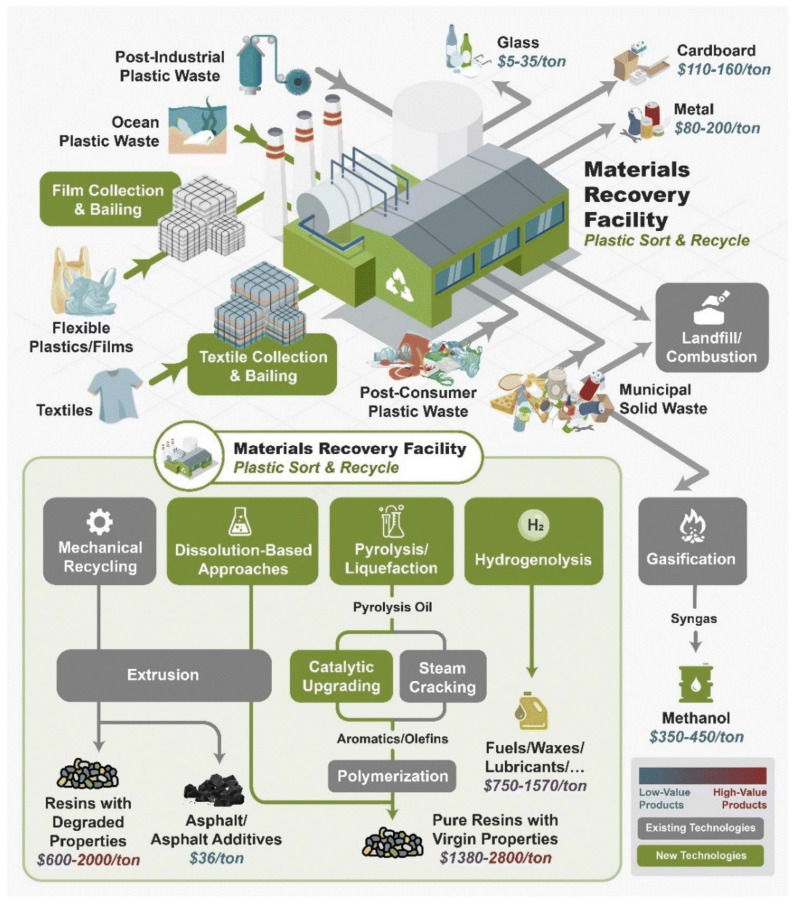
Summary of current management system of waste plastics. Reproduced from [124] with permission from the Royal Society of Chemistry.

The current scenario of plastic waste management is summarized in Figure 7. Sorting plastic waste into distinct streams via the material recovery facility (MRF) is the first stage in the recycling of plastic [124]. Glass, metals, cardboard, and plastics etc., are sorted by the MRF, which then bails them out and sells them to a downstream recycler. Postindustrial waste (PIW), postconsumer waste (PCW), plastics included in municipal solid waste (MSW), and ocean plastics are the four categories into which plastic wastes are separated. Compared to the other forms of plastic waste, PIW usually has a more homogeneous composition and less impurities. PIW is frequently recycled by the industry using closed-loop recycling techniques. 

There are various technologies available today for the chemical recycling of plastics. One approach involves thermal degradation, resulting in a liquid known as pyrolysis oil [125]. This oil can be converted into aromatics and olefins using either steam cracking or a catalytic upgrading process. This process yields aromatics and olefins that can be reused to manufacture new recycled plastics with properties identical to virgin plastics. Multilayer plastics can be processed using dissolution-based recycling methods, producing pure plastic flakes that can be re-extruded into recycled plastic resins. Polyesters and polycarbonates can be chemically or enzymatically broken down into their monomers through methanolysis and other techniques [126]. These monomers can then be reused to recreate the polymers. Plastics can also be gasified to produce synthesis gas, which can be utilized to manufacture methanol or transportation fuels [127,128]. Methanol can subsequently be converted into aromatics and olefins. Additional plastic recycling methods include hydrogenolysis to produce lubricants and oils, functionalizing plastics, and creating plastic alloys, among others [129,130].

The need for sustainable polymer recycling technologies has never been more urgent as the world grapples with the growing environmental crisis caused by plastic waste. The conventional recycling methods frequently fall short, resulting in large quantities of plastic ending up in landfills and oceans, causing significant ecological harm. Sustainable recycling technologies, including chemical recycling, enzymatic breakdown, and advanced dissolution processes, present promising solutions by allowing plastics to be recovered and reused without compromising their quality. These innovative methods can turn waste into valuable resources, reduce reliance on fossil fuels, and decrease greenhouse gas emissions. Embracing sustainable polymer recycling technologies can help establish a circular economy, ensuring that plastics are continually reused and repurposed, thus minimizing their environmental impact and conserving natural resources for future generations. Some of the major methods towards sustainable polymer recycling are:

### 5.1. Mechanical Recycling 

**Figure 8:**
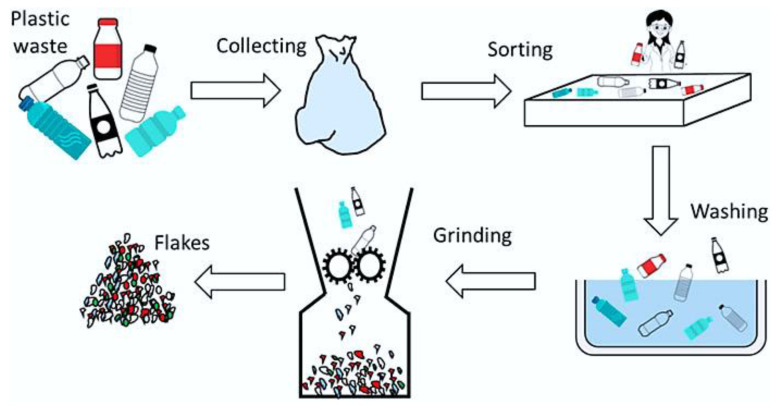
Mechanical recycling for plastic wastes. Image source: [134].

Mechanical recycling [131,132,133] is a key technology in the sustainable management of plastic waste, offering a relatively simple and cost-effective method for converting used plastics into new products. While it has some limitations, advancements in sorting and processing technologies continue to improve its efficiency and effectiveness, making it an integral part of the circular economy for plastics. The process is detailed in Figure 8. 

The process begins with the collection of plastic waste from various sources such as households, industries, and commercial establishments. Once collected, the plastics undergo a sorting phase where they are categorized by type and color, either manually or through automated systems like near-infrared (NIR) spectroscopy, which identifies plastics based on their spectral properties. The sorted plastics are then cleaned in wash tanks to remove contaminants such as dirt, food residues, adhesives, and labels, followed by drying to eliminate any moisture. Next, the cleaned plastics are shredded or ground into smaller pieces known as flakes or granules, which makes handling and further processing more manageable. These flakes are then subjected to separation techniques like density separation, where plastics are separated based on their buoyancy in different liquids, and electrostatic separation, which uses electrical charges to distinguish plastics by their dielectric properties. The purified plastic flakes are then melted in extruders, which are machines that heat and mix the plastic while forcing it through a screw barrel. During the extrusion process, the molten plastic is filtered to remove any remaining impurities. The filtered molten plastic is then extruded through a die to form continuous shapes, typically strands, which are subsequently cooled and cut into pellets. These pellets which are also known as regrind or regranulate, serve as raw material for manufacturing new plastic products. The final stage involves using the recycled pellets in manufacturing processes such as injection molding, blow molding, and extrusion etc. For instance, to create reshaped parts, the pellets are fed into another extruder or a 3D printer. In the extrusion process, the pellets are melted again and extruded into molds to form specific shapes. In 3D printing, the molten plastic is deposited layer by layer to build up the desired object. One of the prominent developments in this regard is the integration of material extrusion filament and 3D printing techniques [135]. These methods are not only efficient but also sustainable, allowing for the upcycling of industrial thermoplastic polymers into high-value products [135,136]. Recent research has focused on enhancing the mechanical properties and thermal stability of recycled filaments to match or exceed those of virgin materials [137]. Innovations in additive manufacturing processes, such as the development of closed-loop recycling systems and the use of advanced composite materials have further expanded the capabilities of 3D printing. There are also advancements in optimizing the extrusion process to reduce energy consumption and improve the quality and consistency of the output. Thus, the mechanical recycling process effectively recycles plastic waste into new usable products, contributing to environmental sustainability by reducing the need for virgin plastic production and minimizing plastic waste in landfills.

### 5.2. Chemical Recycling 

Chemical recycling [138,139,140] of polymers involves breaking down plastic materials into their constituent monomers using chemical processes. Unlike traditional mechanical recycling, which typically downgrades the quality of plastics with each cycle, chemical recycling allows for the regeneration of high-quality polymers that can be used to manufacture new products with properties similar to virgin materials. This approach offers several advantages including the ability to recycle mixed or contaminated plastics that are difficult to process through mechanical means, as well as the potential to recycle plastics that are currently nonrecyclable. Sustainable chemical recycling technologies include processes such as depolymerization, pyrolysis, and gasification, which can convert a wide range of plastic waste streams into valuable feedstocks for the production of new plastics, chemicals, and fuels. A summary of these techniques is given in Table 2. By enabling the closed-loop recycling of polymers, the sustainable chemical recycling technologies play a crucial role in reducing plastic waste, conserving resources, and minimizing environmental pollution.

Apart from this, recent years have seen the development of a novel strategy by researchers to get around the challenge of recycling thermosets by adding chemical linkers to the organic matrix to change it in a way that facilitates the materials’ breakdown while maintaining their mechanical qualities. 

**Figure 9:**
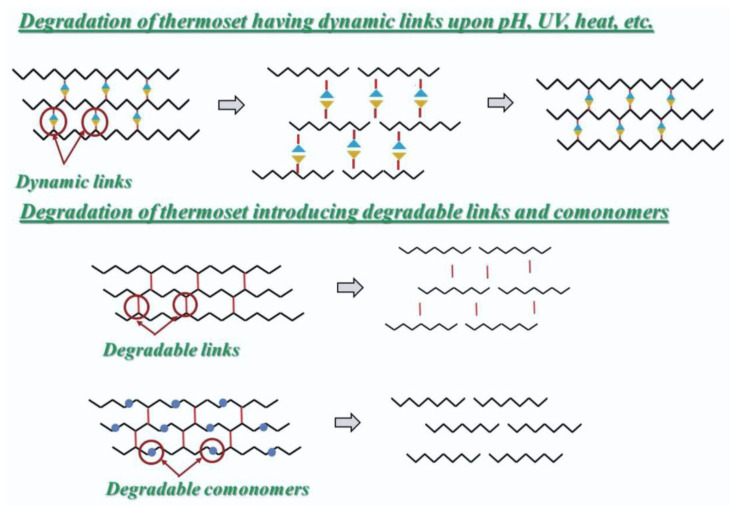
Schematic diagram showing the perspective in developing the thermoset with built-in recyclability. Image reproduced from the reference [141].

The concept involves adding degradable crosslinkers or changing permanently crosslinked structures into dynamic ones so that they can undergo exchange reactions of cleavable links to undergo de- and recrosslinking (Figure 9). These last dynamic bonds are stimuli responsive to heat, irradiation, and acid conditions etc. The fiber-reinforced polymer-composites in which fibers could be readily retrieved after the degradation of the resins are prepared using thermosets with cleavable linkages. Since the dynamic covalent bond strategy allows for direct reshaping, recycling, and reprocessing, thermoset composites can also be made using traditional methods used for thermoplastic matrices such as injection molding and hot pressing etc. In contrast, the presence of degradable crosslinkers forces recycled polymer to be resynthesized or used in low-performing applications [141].

### 5.3. Enzymatic Recycling 

**Figure 10:**
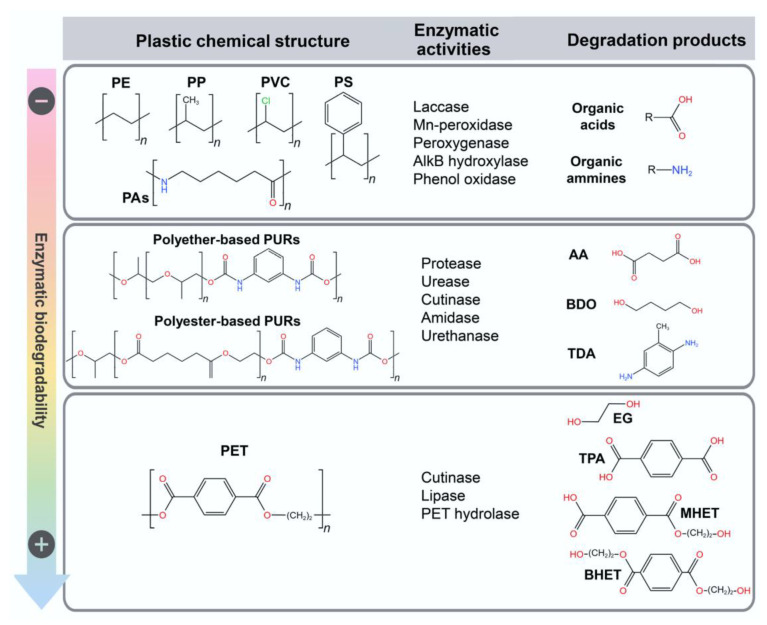
Schematic diagram showing various enzymes involved in the degradation of different types of traditional petroleum-based plastics. Image reproduced from the reference [146].

Enzymatic recycling [142,143,144,145] of polymers represents a promising frontier in the quest for environmentally friendly plastic waste management. This innovative approach utilizes enzymes, biological catalysts, to break down complex polymer structures into their constituent monomers, facilitating the recycling of plastics into their original building blocks. Figure 10 displays various enzymes involved in the breakdown of plastics forming their byproducts [146]. Where the abbreviation are as follows: PE—polyethylene, PP—polypropylene, PAs—polyamides, PVC—polyvinylchloride, PS—polystyrene, PURs—polyurethanes, AA—adipic acid, BDO—1,4-butanediol, TDA—toluene diamine, PET—polyethylene terephthalate, EF—ethylene glycol, TPA—terephthalic acid, MHET—2-hydroxyethyl terephthalate, and BHET—bis(2-hydroxyethyl) terephthalate. 

Unlike traditional recycling methods, enzymatic recycling operates under mild conditions, minimizing energy consumption and reducing the generation of harmful byproducts. Furthermore, enzymatic recycling holds the potential to recycle a wide range of plastics, including those considered difficult to recycle using conventional mechanical methods. By enabling the closed-loop recycling of plastics and reducing reliance on virgin materials, sustainable enzymatic recycling offers a pathway towards a circular economy, where plastics are continually reused, repurposed, and recycled, thus minimizing environmental impact and promoting resource conservation.

### 5.4. Dissolution-Based Recycling 

**Figure 11:**
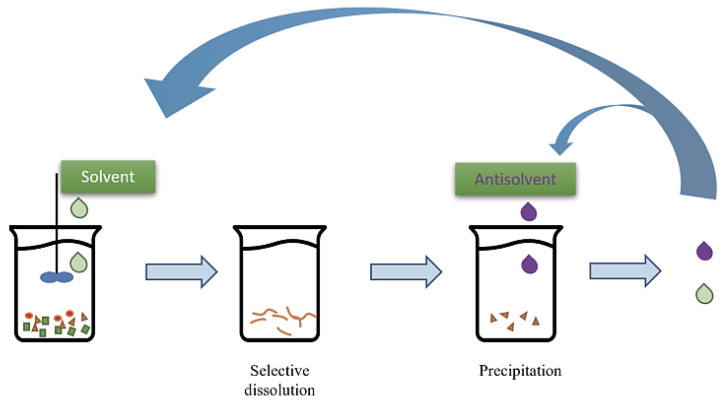
Schematic drawing of the dissolution–precipitation technique. Image reproduced from the reference [149].

Dissolution-based recycling [124,147,148] is an innovative method which involves dissolving plastic materials in a suitable solvent to separate impurities and contaminants, yielding pure polymer solutions that can be used to create new plastic products. Unlike traditional recycling techniques, dissolution-based recycling offers the advantage of processing mixed or contaminated plastics that are challenging to recycle using mechanical methods. Likewise, this process can be tailored to recycle specific types of polymer, enabling the recovery of valuable resources from diverse plastic waste streams. The dissolution–precipitation process using the solvent-non-solvent method is depicted in Figure 11 [149]. The selective dissolution of polymers is carried out at first, which is followed by the precipitation by means of a bad solvent. A lot of commonly used polymers such as PE, PP, PS, PC, PET, and ABC etc., can be processed using this method. 

By promoting the efficient recovery and reuse of plastics, dissolution-based recycling contributes to reducing the environmental burden of plastic pollution and conserving natural resources. As a key component of the circular economy, this method helps close the loop on plastic consumption, offering a sustainable solution to the global plastic waste crisis.

### 5.5. Hybrid Recycling

Hybrid recycling [150,151,152] stands at the forefront of innovative strategies for addressing the complex challenges posed by plastic waste. This approach combines multiple recycling technologies, such as mechanical, chemical, and enzymatic processes, to maximize the recovery and reuse of plastics while minimizing environmental impact. By integrating various recycling methods, hybrid recycling offers a comprehensive solution for handling diverse plastic waste streams, including mixed or contaminated plastics that are difficult to recycle through conventional means. Likewise, hybrid recycling enhances the efficiency and effectiveness of plastic recycling by synergizing the strengths of different processes, such as the ability to depolymerize plastics into monomers and then reprocess them through mechanical means or utilizing enzymatic breakdown to complement chemical recycling. Through its adaptable and versatile nature, hybrid recycling not only promotes resource conservation and waste reduction but also advances the transition towards a circular economy, where plastics are continually recycled, repurposed, and reused, thus fostering sustainability and environmental stewardship.

## 6. Challenges and Future Prospects of Eco-Friendly Polymer Sustainability

Eco-friendly approaches to polymer sustainability offer promising solutions to the pressing environmental challenges posed by conventional plastics. However, these alternatives face a myriad of challenges that must be addressed to realize their full potential. One significant hurdle is the cost associated with producing eco-friendly polymers, which often exceeds that of traditional plastics. Many eco-friendly polymers, such as biodegradable or bio-based ones, often incur higher production costs compared to conventional plastics derived from fossil fuels. This cost disparity is influenced by various factors, including the availability and expense of raw materials, specialized processing equipment, and limited economies of scale. Addressing the cost challenge is pivotal for making eco-friendly polymers more economically viable and competitive in the market.

Another significant challenge lies in ensuring that eco-friendly polymers meet the performance requirements of diverse applications. While sustainable alternatives have made considerable strides in mechanical properties, barrier capabilities, and thermal stability, they may still fall short of the performance characteristics offered by traditional plastics. Achieving performance parity with conventional plastics is essential for garnering widespread acceptance and adoption of eco-friendly polymers across industries such as packaging, automotive, and electronics.

There are also hurdles related to durability and stability, particularly in harsh environmental conditions. Biodegradable polymers, for instance, may degrade prematurely when exposed to moisture, heat, or ultraviolet radiation, compromising their mechanical integrity and functional properties. Developing eco-friendly polymers with enhanced durability and stability while retaining their sustainability attributes is crucial for expanding their applicability in real-world applications and improving end-of-life outcomes. Besides, the challenges in supply chain management and infrastructure further complicate the adoption of eco-friendly polymers. Sustainable sourcing of raw materials and the development of efficient supply chains pose significant challenges, especially for bio-based polymers that rely on renewable biomass feedstocks. Furthermore, the lack of adequate infrastructure for the collection, sorting, and recycling of eco-friendly polymers limits their effective integration into existing waste management systems. Developing robust supply chains and infrastructure to support the entire lifecycle of eco-friendly polymers is imperative for maximizing their sustainability benefits and promoting a circular economy. Lastly, compliance with regulatory requirements and industry standards adds another layer of complexity and challenge to the adoption of eco-friendly polymers. Regulatory frameworks governing the production, use, and disposal of plastics vary across regions and countries, making it challenging for manufacturers to navigate compliance requirements. Likewise, the number of standardized testing methods and certification schemes for eco-friendly polymers complicates the assessment of their environmental impact and performance characteristics. Establishing clear regulatory guidelines and industry standards can help ensure the credibility and transparency of eco-friendly polymer products.

Extended producer responsibility (EPR) is an increasingly recognized strategy for enhancing polymer sustainability by holding manufacturers accountable for the entire lifecycle of their products, including postconsumer waste management. Currently, EPR programs for plastics are implemented with varying degrees of success across different regions. In the European Union, EPR regulations have driven significant improvements in recycling rates and the development of more sustainable packaging solutions [153]. Similarly, countries like Japan and Canada have adopted EPR schemes that encourage producers to design products that are easier to recycle and have lower environmental impacts [154]. However, in many parts of the world, particularly in developing countries, EPR implementation remains fragmented and less effective due to inadequate infrastructure, lack of enforcement, and limited public awareness [155]. In future, the EPR policies are expected to become more comprehensive and stringent with a greater emphasis on circular economy principles. Innovations in tracking and sorting technologies coupled with more robust regulatory frameworks are likely to enhance the effectiveness of EPR schemes. Likewise, increased global collaboration and the harmonization of standards could streamline EPR implementation, ensuring that producers worldwide are incentivized to adopt more sustainable practices [156]. As these initiatives evolve, EPR has the potential to significantly reduce the environmental footprint of polymers, fostering a more sustainable and responsible approach to plastic production and consumption.

Considering the future prospects, one of the key areas of advancement lies in technological innovations aimed at improving the performance, scalability, and cost-effectiveness of eco-friendly polymers. Research and development efforts continue to focus on enhancing the mechanical properties, barrier capabilities, and thermal stability of sustainable alternatives to traditional plastics. Besides, innovations in processing techniques such as 3D printing and advanced molding technologies are expanding the application range of eco-friendly polymers across various industries, including packaging, construction, automotive, and electronics.

Besides, increasing consumer awareness and demand for sustainable products are driving market incentives for eco-friendly polymers. Consumers are becoming more conscious of the environmental impact of their purchasing decisions and are actively seeking out products that align with their values. This shift in consumer preferences is prompting businesses to invest in sustainable solutions and incorporate eco-friendly polymers into their product offerings. As consumer demand for sustainable products continues to grow, manufacturers will be under increasing pressure to adopt environmentally responsible practices and prioritize the use of eco-friendly materials throughout their supply chains.

Considering the technological and consumer-driven advancements, regulatory support for sustainability initiatives is also shaping the future prospects of eco-friendly polymer sustainability. Governments around the world are implementing policies and regulations aimed at reducing plastic pollution, promoting recycling, and incentivizing the use of eco-friendly materials. These regulatory frameworks create a more conducive environment for the adoption of eco-friendly polymers by providing financial incentives, tax breaks, or subsidies for sustainable practices. Likewise, industry standards and certification schemes for eco-friendly polymers are evolving to ensure the credibility and transparency of sustainable products further bolstering their market acceptance and uptake.

The future of eco-friendly polymer sustainability holds great promise for addressing the environmental challenges associated with traditional plastics. With continued technological advancements, growing consumer demand, and supportive regulatory frameworks, eco-friendly polymers are poised to play a pivotal role in reshaping the plastics industry and promoting environmental stewardship on a global scale. By overcoming existing barriers and capitalizing on future opportunities, eco-friendly polymers have the potential to drive positive change and contribute to a more sustainable and circular economy for generations to come.

## 7. Conclusions

The pursuit of enhancing polymer sustainability through eco-conscious strategies is both an urgent necessity as well as a promising frontier. This review highlights how the utilization of bio-based polymers, biodegradable polymers, advancements in recycling technologies, and adoption of green chemistry principles etc., can mitigate the problems existing in the current scenario. The integration of life cycle assessments strategies could further underscore the importance of evaluating the environmental impact of polymers from production to disposal. Further innovative research and interdisciplinary collaboration are essential for overcoming the current challenges and accelerating the transition to sustainable polymer practices. By prioritizing eco-conscious strategies, the polymer industry can significantly reduce its environmental footprint and thereby contribute to a more sustainable future. Continued investments in this field together with supportive policies and public awareness can further facilitate the adoption of sustainable polymers and can ensure a long-term environmental stewardship.

## Figures and Tables

**Table 1:** Summary of comparison between natural and synthetic biodegradable polymers.

	Natural Biodegradable Polymers	Synthetic Biodegradable Polymers
**Source**	Renewable biological sources (plants, animals, microorganisms)	Synthetic processes, renewable feedstocks, sometimes petroleum-based
**Examples**	Cellulose, chitosan, collagen, gelatin	PLA, PCL, PHAs, polyanhydrides, PVA
**Properties**	Biocompatible, nontoxic, lower mechanical strength, and thermal stability	Customizable properties, consistent quality, tailored mechanical, and thermal properties
**Production Methods**	Extraction, purification, chemical, or enzymatic modification	Polymerization (ring-opening, polycondensation), bacterial fermentation
**Applications**	Food packaging, medical applications, agriculture, textiles	Packaging, medical devices, drug delivery, agriculture, consumer goods
**Advantages**	High biocompatibility, nontoxicity, renewable sources, easier biodegradation	Customizable, consistent quality, wide range of applications
**Disadvantages**	Quality variability, limited property customization, potential allergenicity	Higher production costs, potential use of toxic intermediates, specific biodegradation conditions
**Biodegradability**	Often readily biodegradable due to natural enzyme activity	Biodegradability depends on environmental conditions
**Environmental Impact**	Generally lower carbon footprint, promotes sustainable use of resources	Can be designed to have minimal environmental impact, but dependent on production methods
**Economic Considerations**	Potential for lower production costs but variability in quality and supply	Higher production costs but more consistent performance and quality

**Table 2:** A comparison of various chemical recycling techniques such as depolymerization, pyrolysis, and gasification.

Feature	Depolymerization	Pyrolysis	Gasification
**Process**	Breaks down polymers into monomers through chemical reactions.	Breaks down polymers into smaller molecules through high temperature and absence of oxygen.	Converts organic materials into syngas through high temperature and controlled oxygen.
**Feedstock**	Various plastic polymers.	Plastic waste, biomass, and organic materials.	Biomass, coal, and organic waste.
**Products**	Monomers suitable for polymer production.	Pyrolysis oil, gas, and char.	Syngas.
**Circular Economy**	Facilitates closed-loop recycling of plastics, reducing reliance on virgin materials.	Can process mixed or contaminated plastics that are difficult to recycle mechanically.	Offers potential for energy recovery from waste materials, contributing to waste-to-energy initiatives.
